# Akt-Activated Endothelium Increases Cancer Cell Proliferation and Resistance to Treatment in Ovarian Cancer Cell Organoids

**DOI:** 10.3390/ijms232214173

**Published:** 2022-11-16

**Authors:** Jessica Hoarau-Véchot, Morgane Blot-Dupin, Léa Pauly, Cyril Touboul, Shahin Rafii, Arash Rafii, Jennifer Pasquier

**Affiliations:** 1 Department of Genetic Medicine and Obstetrics and Gynecology, Genetic Intelligence Laboratory, Weill Cornell Medicine-Qatar, Education City, Qatar Foundation, Doha P.O. Box 24144, Qatar; 2 Faculté de Médecine de Créteil UPEC—Paris XII, Service de Gynécologie-Obstétrique et Médecine de la Reproduction, Centre Hospitalier Intercommunal de Créteil, 40 Avenue de Verdun, 94000 Créteil, France; 3 Institut National de la Santé et de la Recherche Médicale (INSERM), Centre National de la Recherche Scientifique (CNRS), UMR_S 938, Centre de Recherche Saint-Antoine, Team Cancer Biology and Therapeutics, Institut Universitaire de Cancérologie, Sorbonne Université, 75012 Paris, France; 4 Department of Obstetrics and Gynecology, Hôpital Tenon, Assistance Publique Des Hôpitaux de Paris, GRC-6 UPMC, Université Pierre et Marie Curie, 75005 Paris, France; 5 Department of Genetic Medicine, Weill Cornell Medicine, New York, NY 10021, USA

**Keywords:** ovarian cancer, tumor microenvironment, cell–cell interactions, endothelial cells, 3D model

## Abstract

Ovarian cancer (OC) is a heterogeneous disease characterized by its late diagnosis (FIGO stages III and IV) and the importance of abdominal metastases often observed at diagnosis. Detached ovarian cancer cells (OCCs) float in ascites and form multicellular spheroids. Here, we developed endothelial cell (EC)-based 3D spheroids to better represent in vivo conditions. When co-cultured in 3D conditions, ECs and OCCs formed organized tumor angiospheres with a core of ECs surrounded by proliferating OCCs. We established that Akt and Notch3/Jagged1 pathways played a role in angiosphere formation and peritoneum invasion. In patients’ ascites we found angiosphere-like structures and demonstrated in patients’ specimens that tumoral EC displayed Akt activation, which supports the importance of Akt activation in ECs in OC. Additionally, we demonstrated the importance of FGF2, Pentraxin 3 (PTX3), PD-ECGF and TIMP-1 in angiosphere organization. Finally, we confirmed the role of Notch3/Jagged1 in OCC–EC crosstalk relating to OCC proliferation and during peritoneal invasion. Our results support the use of multicellular spheroids to better model tumoral and stromal interaction. Such models could help decipher the complex pathways playing critical roles in metastasis spread and predict tumor response to chemotherapy or anti-angiogenic treatment.

## 1. Introduction

Serous ovarian cancer (OC) is the eleventh most common cancer worldwide and the fifth leading cause of death among women [1,2]. OC is characterized by late diagnosis (FIGO stages III and IV) resulting in abdominal metastatic spread [3,4]. OC spread is believed to be mainly associated with ascites formation and occurs through peritoneal circulation of floating cancer cells [5]. The mechanism of ovarian cancer cell (OCC) spread is still not well understood. Detached cancer cells float in ascites and form multicellular spheroids [5]. These spheroids interact with the mesothelium in order to form implants in peritoneal organs [6]. Preclinical and clinical observations suggest the role of vascular endothelial growth factor (VEGF) and endothelial cells (ECs) in this process [7,8] but the presence of ECs in ascites and their interaction with OCCs has not been comprehensively studied [9].

ECs are known to participate (through diverse molecular pathways) in most aspects of tumoral biology, from tumor growth to resistance to chemotherapy or targeted therapies [10,11].

In this context, we hypothesized that ECs could be involved in OCC spread.

We engineered stable ECs cultured in cytokine-free serum-free media to act as an Akt-dependent durable vascular niche [12]. The Akt pathway has been shown to be implicated in the pathogenesis of ovarian cancer. Its activation drives cellular growth and survival. In previous studies, our group has shown, similarly to others, that cancer cells could lead to the activation of a normal endothelium. We showed that Akt-activated ECs (E4 + EC) create a pro-tumoral niche through contact-mediated or secreted factors in models such as lymphatic, breast and ovarian cancer [13,14,15,16,17,18,19,20,21].

Current in vitro cancer cell models have major limitations regarding their ability to replicate the complexity of in vivo structures. The recent development of 3D culture technologies addresses this limitation by providing platforms to integrate different constraints [22,23,24,25].

Here, we developed a new model of organized multicellular 3D spheroids to study tumor–endothelial interactions. We demonstrated that, when co-cultured in 3D conditions, E4 + ECs and OCCs formed Akt-dependent organized tumor angiospheres with a core of endothelial cells surrounded by highly proliferating OCCs. We found similar structures in patients’ ascites and showed that ECs were Akt-activated in a cohort of 59 patients. FGF2, Pentraxin 3 (PTX3), PD-ECGF and TIMP-1 played a role in angiosphere organization. We demonstrated the resistance of our angiosphere to classical chemotherapy or anti-angiogenic therapy. Finally, we confirmed the role of Notch3/Jagged1 in OCC–EC crosstalk in relation to OCC proliferation and peritoneal invasion.

## 2. Results

### 2.1. Akt Activation in ECs Is Mandatory for the Formation of Organized Angiospheres 

**Figure 1:**
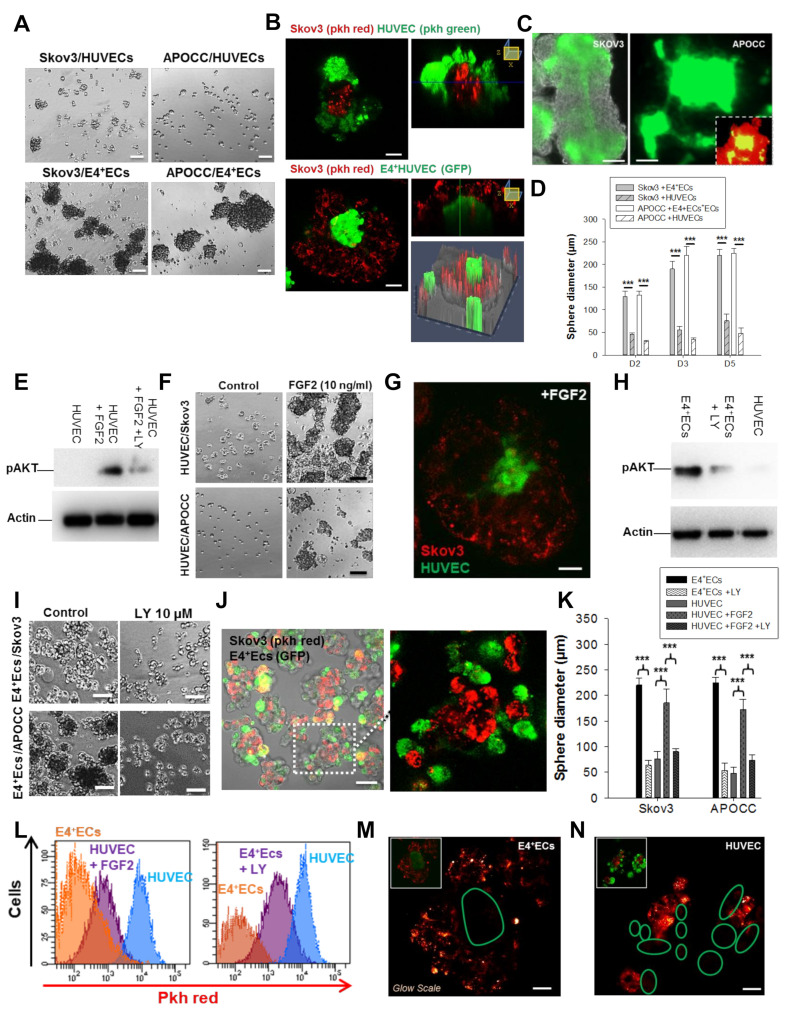
(**A**) Spheroids of SKOV3 and APOCC co-cultured with HUVECs or E4 + ECs were grown in 3D media for 6 days. Scale bar, 50 µm. (**B**) SKOV3 cells were stained with PKH red prior to being co-cultured with HUVECs stained with PKH green (top panel) or eGFP-E4 + ECs (bottom panel). Z-X reconstitution is shown on the right. Top scale bar, 10 µm. Bottom scale bar, 30 μm. (**C**) SKOV3 (left panel) and APOCC (right panel) were co-cultured with eGFP-E4 + ECs. eGFP fluorescence was revealed in epifluorescence. Scale bar, 20 µm. (**D**) Spheroids of SKOV3 cells and APOCC co-cultured with HUVECs or co-cultured with E4 + ECs were grown for 6 days. Spheroid diameters were measured at D2, D3 and D5. *p* < 0.001 (***). (**E**) Western blot of pAkt on untreated HUVECs, HUVECs treated with FGF2 and HUVECs treated with FGF2 + LY 294002. Actin was used as control. (**F**) Spheroids of SKOV3 cells and APOCC co-cultured with HUVECs pre-treated or not with FGF2 (10 ng/mL) were grown for 6 days. Scale bar, 50 µm. (**G**) SKOV3 cells were stained with PKH red prior to being co-cultured with HUVECs stained with PKH green and treated with FGF2 (10 ng/mL). Scale bar, 30 μm. (**H**) Western blot of pAkt on E4 + ECs, E4 + ECs treated with LY 294,002 and HUVECs. Actin was used as control. (**I**) Spheroids of SKOV3 cells and APOCC co-cultured with E4 + ECs pre-treated or not with LY 294,002 (10 µM) were grown for 6 days. Scale bar, 50 µm. (**J**) SKOV3 cells were stained with PKH red prior to being co-cultured with eGFP-E4 + ECs treated with FGF2 (10 ng/mL). Scale bar, 15 μm. (**K**) The bar graph represents the sphere diameters at D6 for the different co-culture conditions of SKOV3 and APOCC with HUVECs or E4 + ECs. (**L**) SKOV3 cells were stained with PKH red prior to being co-cultured with HUVEC, HUVEC treated with FGF2 (10 ng/mL), E4 + ECs or E4 + ECs treated with LY 294,002 (10 µM). PKH level was evaluated by flow cytometry. (**M**,**N**) SKOV3 cells were stained with PKH red prior to being co-cultured with eGFP-E4 + ECs (**M**) and with HUVECs stained with PKH green (**N**). Pictures are shown in glow scale. Scale bar, 50 µm (**M**) and 20 µm (**N**). Green circles represent the location of eGFP-E4 + ECs or HUVECs stained with PKH green. (**B**,**G**,**J**,**M**,**N**): pictures were taken via confocal imaging.

To investigate the role of Akt-activated ECs in OC, we used HUVECs (human umbilical vein endothelial cells) and Akt-activated ECs (E4 + EC) obtained by transfection of HUVECs with the E4orf1 gene [14]. To test the interaction between endothelial and cancer cells, we co-cultured SKOV3 or a primary ovarian cancer cell line APOCC on ultra-low attachment plates [23]. E4 + ECs contributed to significant spheroid growth and HUVECs used in similar seeding densities were unable to contribute to sphere expansion (Figure 1A). In confocal analysis, the spheres with E4 + ECs displayed a core of endothelial cells surrounded by cancer cells. In contrast, HUVEC cells surrounded cancer cells and were not able to penetrate the spheres (Figure 1B). Within the spheres, endothelial cells organized themselves and often formed tubular-like structures with cancer cells expanding around them (Figure 1C). The angiospheres formed with E4 + ECs were larger (at D6, 220 ± 13.6 µm for SKOV3 and 225 ± 10.2 µm for APOCC) compared to the ones with HUVEC (at D6, 75.7 ± 15.1 µm for SKOV3 and 48.2 ± 12.1 µm for APOCC) (Figure 1D). We previously demonstrated that FGF2 induced Akt phosphorylation in endothelial cells leading to their activation and their resistance to anti-VEGF drugs such as bevacizumab [17]. We confirmed Akt phosphorylation in HUVECs treated with FGF2 (10 ng/mL) by Western blot analysis and confirmation of its inhibition by the Akt inhibitor LY294002 (Figure 1E and Supplementary Figure S1). Concordantly, treatment of HUVECs with FGF2 before sphere formation induced an increase in sphere numbers and size (Figure 1F).

FGF2 treatment also modified the structure of HUVEC angiospheres, resulting in a central core of HUVECs surrounded by cancer cells, as observed with E4 + ECs (Figure 1G). To demonstrate the primordial role of Akt phosphorylation in the constitution of the 3D structures, we inhibited Akt activation in the E4 + ECs before 3D sphere formation using LY294002 (Figure 1H, Supplementary Figure S1). LY294002 treatment did not compromise endothelial cell survival, however, the ability of endothelial cells to produce spheres in 3D media was significantly reduced. In co-cultures, spheres were still forming but the treatment resulted in abrogation of cancer sphere expansion (Figure 1I) and resulted in the “HUVEC-like” organization of spheres with endothelial cells surrounding cancer cells (Figure 1J). Sphere sizes reflecting OCC proliferation were similar to the ones formed with HUVECs for both cancer cells lines tested in the presence of LY294002 (at D6, 63.22 ± 9.2 µm for SKOV3 and 52.6 ± 15.8 µm for APOCC) (Figure 1K).

We used the PKH dilution assay to evaluate cancer cell proliferation within angiospheres [26]. We confirmed that cancer cell line proliferation resulted in a dilution of PKH red at D3 and D5 (Supplementary Figure S2). We observed lower PKH red levels in SKOV when co-cultured with E4 + ECs compared to co-culturing with HUVECs. When we treated SKOV/E4 + ECs in co-cultures with LY294002, PKH red increased in SKOV;, when we treated SKOV/HUVECs in co-cultures with FGF2, PKH red was lower in SKOV (Figure 1L). Under confocal microscopy, PKH red staining in APOCC was higher at the periphery of the sphere and decreased toward the E4 + EC core, suggesting a role of E4 + ECs in cancer cell proliferation (Figure 1M). In HUVEC angiospheres, the intensity of PKH staining was consistent through OCCs (Figure 1N). OCCs are the cells that contribute the most to sphere diameter increases. Overall, we showed that Akt-activated endothelial cells formed a central core surrounded by ovarian cancer cells. The central core seemed to provide angiocrine cues to promote cancer cell growth.

### 2.2. Endothelial Jagged1 Is Mandatory for OCC Proliferation

**Figure 2:**
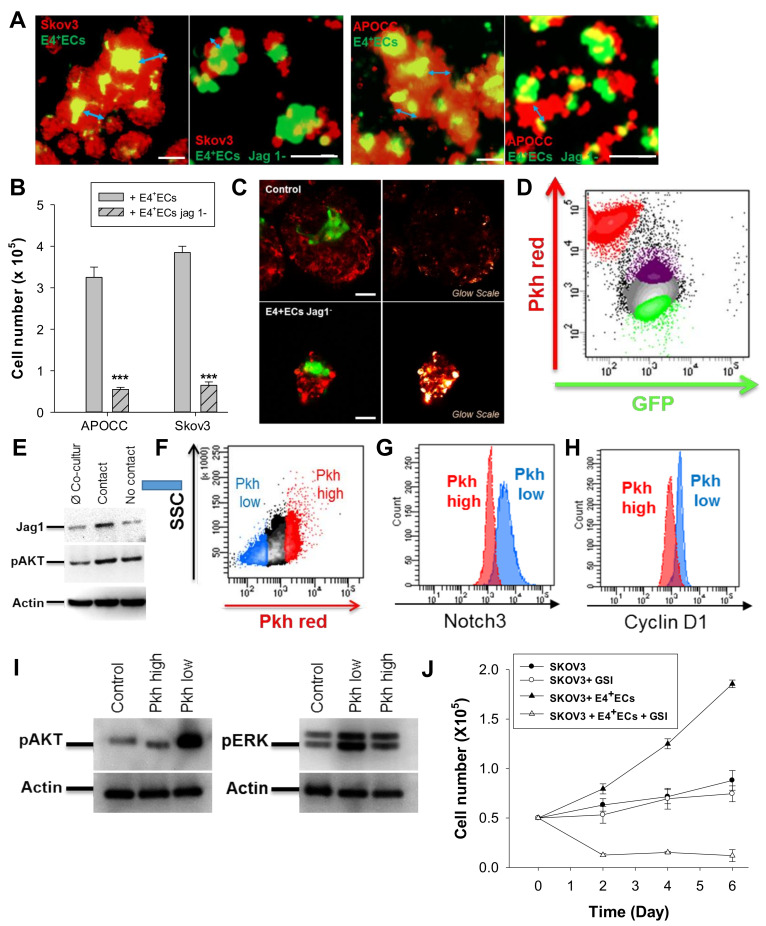
(**A**) Spheroids of SKOV3 and APOCC were stained with PKH red prior to being co-cultured in 3D media with eGFP-E4 + ECs or eGFP-SH-jag1-E4 + ECs. Pictures were taken via confocal imaging. Scale bar, 100 μm. (**B**) The bar graph represents the sphere diameters at D6 for co-cultures of OCCs (SKOV3 and APOCC) and eGFP-E4 + ECs or eGFP-SH-jag1-E4 + ECs. *p* < 0.001 (***). (**C**) SKOV3 cells were stained with PKH red prior to being co-cultured in 3D media with eGFP-E4 + ECs (top pictures) or eGFP-SH-jag1-E4 + ECs (bottom pictures). Pictures with glow scales were taken via confocal imaging. Scale bar, 50 µm (**D**) SKOV3 cells were stained with PKH red prior to being co-cultured in 3D media with eGFP-E4 + ECs. eGFP-E4 + ECs in contact with SKOV3 (purple population) or not in contact (green population) were sorted at D6. (**E**) Western blotting for Jag1 and pAkt was performed on sorted E4 + ECs. Actin was used as control. (**F**) SKOV3 cells were stained with PKH red prior to being co-cultured in 3D media with eGFP-E4 + ECs. After 6 days of co-culture, PKH low SKOV3 (blue population) and PKH high SKOV3 (red population) were sorted. g-h Notch3 (**G**) and Cyclin D1 (**H**) were evaluated in the two SKOV3 populations sorted in F by flow cytometry. (**I**) Western blotting for pERK and pAkt was performed on sorted SKOV3. Actin was used as control. (**J**) Spheroids of SKOV3 with or without E4 + ECs were cultured in the presence or not in the presence of GSI for 6 days. Every 2 days, SKOV3 cells were counted.

We described the instructive function of endothelial Jagged1 in supporting self-renewal and the regenerative capacity of HSCs in the adult BM vascular niche [27], as well as its function in inducing aggressiveness and chemoresistance in lymphoma [13], breast cancer cells [18,19] and, more recently, in ovarian cancer [21]. To investigate the specific effect of endothelial Jagged1, we used an E4 + ECsjag1KD cell line with inhibited Jagged1 expression (Supplementary Figure S3). E4 + ECsjag1KD significantly reduced sphere diameters (Figure 2A,B). The PKH red signal from OCCs was concordant with the absence of proliferation in E4 + ECsjag1KD angiospheres (Figure 2C). Similarly, the number of invading endothelial tubes observed inside the sphere was significantly reduced (Supplementary Figure S4).

To confirm the role of the Notch pathway in OCC proliferation, we used a trogocytosis assay to identify E4 + ECs in direct contact with OCCs [28]. After 3 days of co-culture with PKH red-stained SKOV3, we observed an increased expression of Jagged1 in E4 + ECs with an uptake of PKH staining from PKH red-stained SKOV3 (Figure 2D,E and Supplementary Figure S5). We verified that Akt phosphorylation was similar in all E4 + EC populations (with or without PKH staining) (Figure 2E). We then sorted the OCCs based on their PKH intensity after 5 days of co-culture (Figure 2F) and the PKHlow dividing cells displayed higher Notch3 staining associated to a higher expression of CyclinD1, as well as higher phosphorylation of the Akt and ERK1/2 pathway (Figure 2G–I and Supplementary Figure S5). Concordantly, the Notch gamma secretase inhibitor (GSI, 20 µM) inhibited the proliferation of OCCs in the angiospheres while having no effect on SKOV3 alone (Figure 2J and Supplementary Figure S5).

### 2.3. Angiocrine Secreted Factors Induce Angiosphere Core-Based Organization

**Figure 3:**
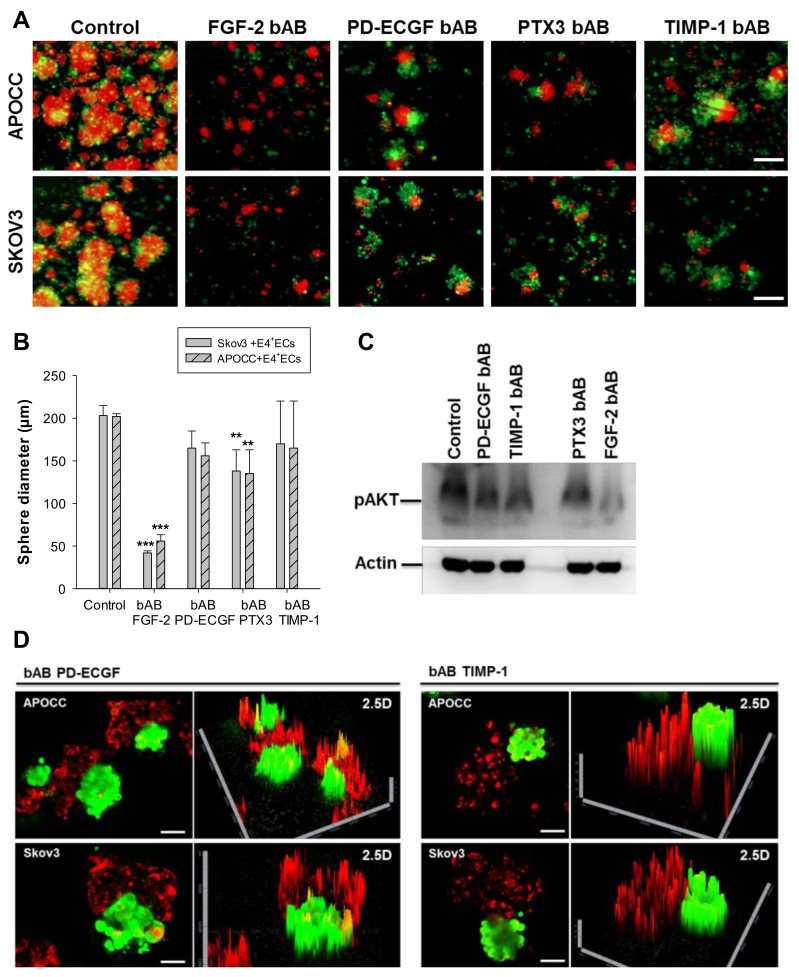
(**A**) Spheroids of SKOV3 and APOCC were stained with PKH red prior to being co-cultured in 3D media with eGFP-E4 + ECs. Spheroids were cultured for 6 days in the presence of different blocking antibodies (FGF-2 bAB, PD-ECGF bAB, PTX3 bAB and TIMP-1 bAB). Scale bar, 250 µm. (**B**) The bar graph represents the sphere diameters at D6 for the different conditions of the co-cultures of OCCs (SKOV3 and APOCC) and eGFP-E4 + ECs. *p* < 0.01 (**), *p* < 0.001 (***). (**C**) Western blotting for pAkt was performed on E4 + ECs treated or not treated with the different bABs. Actin was used as control. (**D**) Spheroids of SKOV3 and APOCC were stained with PKH red prior to being co-cultured in 3D media with eGFP-E4 + ECs. Spheroids were cultured for 6 days in the presence of different blocking antibodies (PD-ECGF bAB and TIMP-1 bAB). Confocal imaging was performed on the spheroids and 2.5D reconstructions are displayed. Scale bar, 200 µm.

After ruling out the role of Jagged1 in angiosphere organization, we investigated the influence of secreted cytokines. We performed a cytokine array on the supernatant of E4 + ECs and HUVECs treated or not treated with LY294002 and/or FGF2 to identify the angiocrine repertoire of activated endothelia (Table 1). Four cytokines were secreted by E4 + ECs and Akt-activated HUVECs: FGF2, Pentraxin 3 (PTX3), PD-ECGF and TIMP-1 (Table 1, orange highlight). To investigate their roles, we used a blocking antibody at D0 of sphere formation and renewed it at each medium replacement (Figure 3A).

While the four bABs (FGF2-bAB, PD-ECGF-bAB, PTX3-bAB and TIMP-1-bAB) were able to disrupt E4 + ECs/OCC sphere organization and reduce the number of spheres in the culture, only FGF-2 bAB and PTX3 bAB had a significant effect on the diameter of the sphere at D3 (Figure 3B). We performed Western blot analysis for pAkt on E4 + ECs treated or not treated with the different bABs (Figure 3C and Supplementary Figure S6). Interestingly, only PTX3 and FGF-2 bABs were able to significantly reduce the level of Akt phosphorylation, explaining why the sphere diameters were reducing in the presence of these two bABs. This confirms that Akt phosphorylation is mandatory for the formation of larger spheres. Without significantly impacting the size of the sphere, TIMP-1 and PD-ECGF bABs affected their organization. In the presence of PD-ECGF and TIMP-1 bABs, E4 + ECs and OCCs formed spheres but never mixed closely (Figure 3D). In contrast, confocal analysis of the spheres in the presence of FGF-2 and PTX3 bABs showed un-organized small spheres (Supplementary Figure S7).

### 2.4. Angiospheres Are Associated with Increased Resistance to Chemotherapy and Anti-Angiogenic Treatment

**Figure 4:**
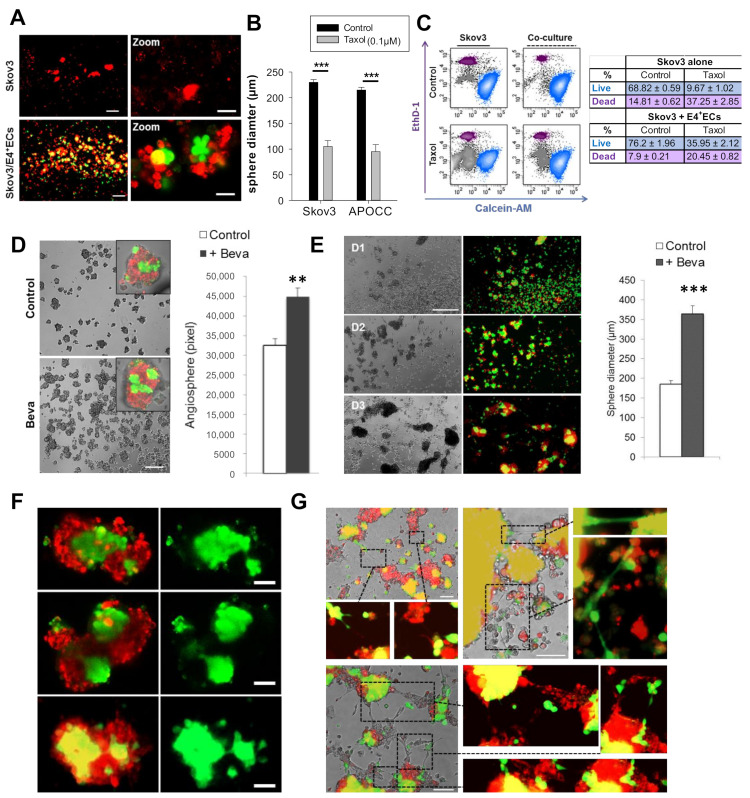
(**A**) SKOV3 cells were stained with PKH red prior to being co-cultured in 3D media with eGFP-E4 + ECs. At D6, spheroids were treated with 0.1 µM of taxol for 48 h. SKOV3: Scale bar, 100 µm; Zoom SKOV3: Scale bar, 50µm; SKOV3/E4^+^ECs: Scale bar, 500 µm; Zoom SKOV3/E4^+^ECs, Scale bar, 250 µm (**B**) The bar graph represents the sphere diameters after 48 h of treatment. (**C**) A live/dead assay was performed on spheres treated or not treated with taxol (0.1 µM). The percentage of live cells (blue population) and dead cells (purple population) is represented in the table on the right. (**D**) SKOV3 cells were stained with PKH red prior to being co-cultured in 3D media with eGFP-E4 + ECs in the presence or not in the presence of bevacizumab (Beva). The bar graph represents the number of spheres at D5. *p* < 0.01 (**). Scale bar 300 µm (**E**) SKOV3 cells were stained with PKH red prior to being co-cultured in 3D media with eGFP-E4 + ECs. Bevacizumab was only added once at D0 and not renewed at all. The bar graph represents the sphere diameters as total pixels at D5. Scale bar, 500µm. *p* < 0.001 (***). (**F**,**G**) Confocal picture of spheres from the experiment in E focusing on the sphere core. Scale bar, 50 µm (**F**) and endothelial tube formation of intra- and inter-spheres. Scale bar, 100 µm. (**G**).

We assessed chemoresistance in our 3D culture setting. A 48 h treatment with 0.1 µM of taxol disrupted SKOV3 sphere structures, while angiospheres maintained a central core of E4 + ECs (Figure 4A).

The angiosphere diameters were, however, significantly smaller when treated with taxol (105 ± 11.5 µm and 95 ± 13.5 µm vs. SKOV3 and APOCC, respectively) (Figure 4B). The presence of E4 + ECs increased SKOV3 survival after 5 days of treatment (live cells: 9.67% ± 1.02 in the control, 35.95 ± 2.12 in the angiospheres) (Figure 4C).

We also investigated the effect of bevacizumab in our model. When bevacizumab was added daily for 5 days, angiosphere structures were not affected and the number of spheres increased significantly (Figure 4D). This could be explained by the autocrine loop based on FGF2 secretion demonstrated by our team [17]. Interestingly, when bevacizumab was added only at D0, the size of angiospheres and the EC core increased, suggesting EC proliferation (Figure 4E,F). The number of tubes between the spheres increased as well (Figure 4G).

### 2.5. Angiospheres Participate in Peritoneal Invasion In Vivo

**Figure 5:**
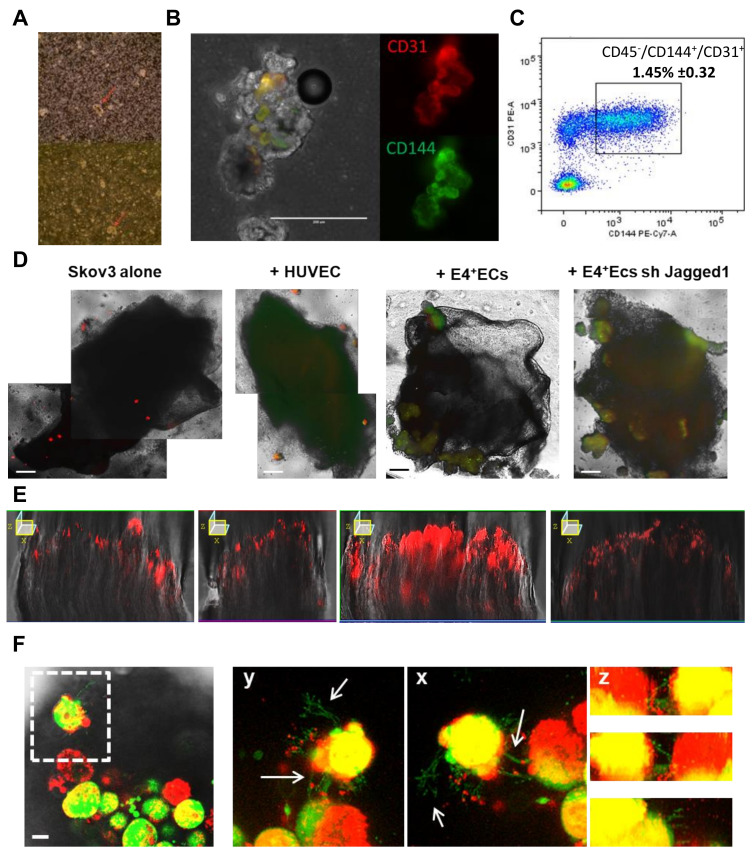
(**A**) Ascites of patients were screened through phase microscopy for spheroid formations (red arrow). (**B**) Spheroids from patient ascites were stained with CD31 and CD144. Scale bar, 200 µm. (**C**) Cells from patient ascites were stained with CD45, CD144 and CD31 and CD45-/CD144+/CD31+ cells were gated. The full gating strategy is available in Supplementary Figure S9. (**D**) SKOV3 cells were stained with PKH red prior to being co-cultured in 3D media with HUVECs stained with PKH green, eGFP-E4 + ECs or eGFP-SH-jag1-E4 + ECs. After 6 days of co-culture, spheroids were put in contact with patient peritonea pieces. Scale bar, 500 µm (**E**) Z-X reconstitution of red fluorescence is shown for each condition. (**F**) Pictures display confocal imagery taken deep in peritonea in contact with spheroids of SKOV3 and eGFP-E4 + ECs. Arrows are pointing at endothelial sprouting coming from eGFP-E4 + ECs. Scale bar, 20 µm.

A major clinical issue in ovarian cancer is the early invasion of the peritoneum by detached cellular aggregates. To investigate the existence of angiospheres in vivo, we first identified cellular aggregates inside EOC-derived patient ascites (Figure 5A, n = 10).

These aggregates contained ECs (CD144+, CD31+) (Figure 5B, n = 3). Using flow cytometry, we determined that endothelial cells (CD45-, CD144+ and CD31+) represented 1.45% ± 0.32 of the cellular population in patients’ ascites (n = 5, Figure 5C).

To investigate whether angiospheres would display higher ability to invade the peritoneum, we cultured SKOV3 spheres and angiospheres (SKOV3/HUVEC, SKOV3/E4 + ECs or SKOV3/E4 + ECsjag1KD) in suspension in serum-free media containing 4% matrigel and peritoneal biopsies (Figure 5D). Confocal Z stack imaging showed that cancer cells from E4 + ECS angiospheres infiltrated the peritoneum deeper compared to HUVEC or E4 + ECsjag1KD angiospheres (Figure 5E). At early time points, sphere adhesion to the peritoneal surface resulted in the formation of web-like structures initiated from endothelial cells toward the peritoneum (Figure 5F). These structures were only observed in mixed spheres with E4 + ECs, and not with HUVECs nor E4 + ECsjag1KD.

### 2.6. Tumor Endothelial Cells Are Akt-Activated

**Figure 6:**
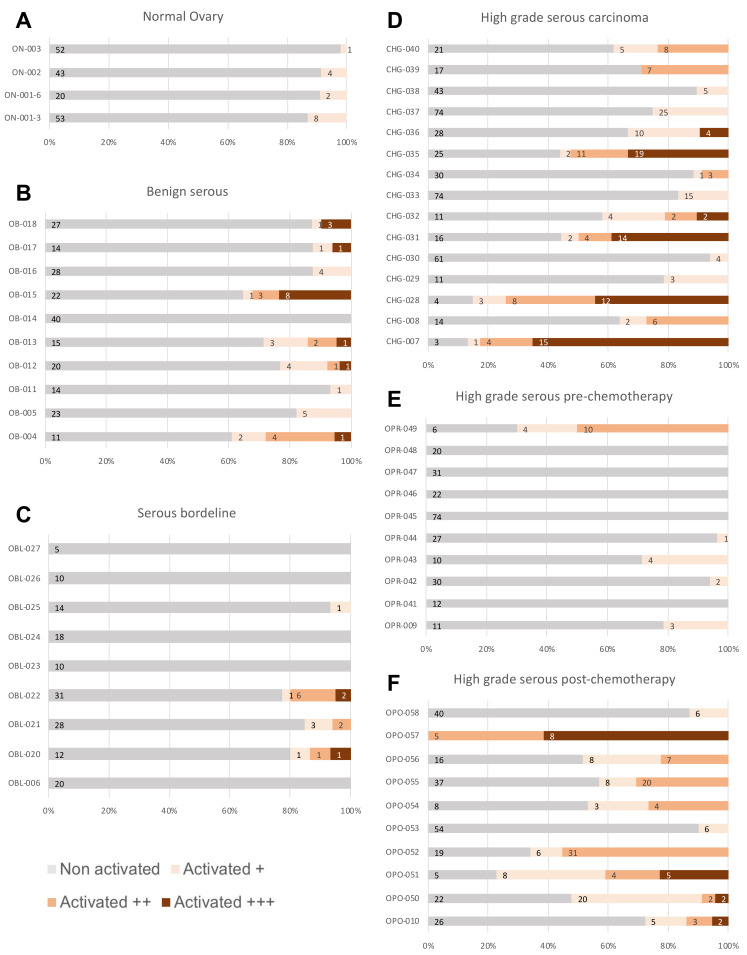
Samples from patients who had a suspected ovarian tumor were collected during debulking surgeries. After the histological material was fixed, sectioned and colored, it was analyzed under microscope. Immunohistochemistry S-P assays were used to investigate the expression of pAkt. The stacked bar graphs show the level of pAkt staining per patient sample identified in normal ovary (**A**), benign serous (**B**), serous borderline tumor (**C**), high-grade serous carcinoma (**D**), high-grade serous pre-chemotherapy carcinoma (**E**) and high-grade serous post-chemotherapy carcinoma (**F**). Vessels were considered as activated +, ++ or +++ depending on the staining intensity.

We investigated the presence of Akt-activated endothelia in vivo. Samples from patients who had a suspected ovarian tumor were collected during debulking surgeries. Immunohistochemistry S-P assays were used to investigate the expression of pAkt. A total of 58 sections were examined (Figure 6 and Supplementary Figure S8) and divided into six different subgroups: normal ovary (ON), serous benign (OB), serous borderline (OBL), high-grade serous carcinoma (CHG), high-grade serous pre-chemotherapy (OPR) and high-grade serous post-chemotherapy (OPO).

We observed a low number of Akt-activated vessels on average in the ON (8.2%) and OBL (7.16%) groups compared to OB (18.8%) and CHG (37%) groups. The presence of a higher number of Akt-activated vessels in OB and CHG groups supports the role of activated endothelia in OC. When we compared sections before and after chemotherapy, we noticed a significantly higher number of Akt-activated vessels after chemotherapy (12.8 vs. 48.4%), implying the role of activated endothelia in resistance to treatment or recurrence through an activated vascular niche as the samples were analyzed after chemotherapy originated from metastasis.

## 3. Discussion

Here, using activated endothelial cells, we created tumor spheres where the interaction between cancer cells and endothelial cells could be modeled. We showed the requirement of an Akt activation in the endothelium for sphere formation and proliferation. Our findings are concordant with the existence of a specific tumor endothelium as determined by gene profiling studies [19,29,30,31,32]. Tumoral ECs produce angiocrine factors promoting tumor growth [33,34], in particular in the context of hypoxia, as found in the central core of tumoral spheres [23]. The “angiocrine switch” (activation of an angiocrine repertoire by activated endothelia) occurs at an early stage of tumor development and contributes to the formation of a pro-tumoral niche. We previously showed the role of such Akt-activated angiocrine endothelia in the stimulation of the autocrine FGF2/FGFR1 loop and its induced resistance to an anti-angiogenic agent [17]. Interestingly, in our study, treatment by chemotherapy and an anti-angiogenic drug in our 3D culture setting provided us with promising results (Figure 4). More research would be necessary in order to study other drugs and evaluate different dosages.

Another team has also shown the role of the perivascular niche in resistance to radiation in medulloblastoma through the activation of the Akt/mTOR pathway in perivascular nestin-expressing stem cells [35].

In addition to the role of Akt, we showed the importance of the Jagged1 pathway to crosstalk in connection with sphere activity independent of formation and expansion. The ovary is the organ with the strongest physiological angiogenesis because of its cyclic corpus luteum [36]. Ovary morphogenesis and angiogenesis are dependent on Notch interactions with ECs. Ovarian cancer cells express Notch1, Notch2, Notch3, Notch4 and Jagged2, while ECs located in the ovary express Jagged1 and Dll1 and Dll4 ligands. VEGF-mediated microvascular growth is controlled by the Notch pathway. [37] In ovarian cancer tissues, Dll4 was positively associated with VEGFR1 expression, and Notch1 was positively correlated with VEGFR2 expression and microvessel density [38]. The comparison of gene expressions in ECs from 10 invasive epithelial ovarian cancers and from five normal ovaries, realized by Lu et al., demonstrated that Jagged1 was over expressed in the ECs of invasive epithelial ovarian cancers compared to normal ovaries [29].

We showed that the secreted cytokines FGF2, Pentraxin 3 (PTX3), PD-ECGF and TIMP-1 could stabilize the angiosphere structure. PTX3 has both pro- and anti-tumor effects. In breast cancer, its level increases under hypoxia and contributes to EMT and stem cell-like trait induction in tumor cells [39,40,41]. PTX3 production in endothelial cells (in vitro and in vivo) was also shown to be induced by HDL through the activation of the PI3K/Akt pathway through G-coupled lysosphingolipid receptors [42].

TIMP-1 was originally characterized as an endogenous inhibitor of metalloproteinases (MMPs) and known for its role in extracellular matrix remodeling, both of which are crucial for tumor invasion and metastasis [43,44,45]. Song et al. reported that TIMP1 activates the FAK-PI3K/Akt and MAPK pathways and plays an important role in colorectal cancer progression and metastasis [46]. More recently, it was reported that TIMP-1 was overexpressed by platinum-resistant TOV-112D and OVSAHO EOC cells. They were able to act as a double-edged sword in the epithelial ovarian cancer microenvironment by altering the response of tumor cells to platinum treatment and indirectly affecting the migration and proliferation of endothelial cells [47]. PD-ECGF is also identified as an angiogenic factor [48,49,50]. Bijnsdorp et al. showed that the exposure of endothelial cells to conditioned media derived from cancer cells with high PD-ECGF expression stimulated their migration and invasion but not their proliferation [50]. It was also reported that tumor-bearing animals had elevated thymidine phosphorylase activity in their ascites and plasma [51,52].

Recently, 3D culture methods have been extensively used to create patient-derived organoids with better modeling of the disease. Such progress could improve our understanding of molecular mechanisms underlying diseases and be used as a platform to develop personalized therapy. In cancer, the use of tumoroids has allowed us to better understand resistance to treatment and better mimic tumor interaction with its microenvironment [53,54]. In 3D cultures, the established organoids maintain tumor heterogeneity, mutational landscape and gene expression and can be used to test patient-specific treatment strategies [55]. It has now become common to study cohorts of patients receiving standard systemic treatment that are being assessed clinically for their response. Survival statistics are then correlated retrospectively with the molecular tumor profile. However, despite the fact that this strategy is helpful in determining the relationship between molecular profile and response, it is time-consuming and requires a large group of patients, which will not serve patients who are in need of effective treatment today. In this context, using tumoroids along with drug sensitivity testing and molecular assessment could be a way of offering personalized anticancer therapy to current cancer patients and therefore improving their treatments.

Additionally, 3D models have been used to better capture the hallmarks of ovarian cancer [56]. Kopper et al. presented a protocol where they were able to derive and expand long-term OC organoids. Using OC tissue from patients, they established 56 organoid lines from 32 patients, representing all main subtypes of OC. These OC organoids, which illustrated intra- and interpatient heterogeneity, recapitulate histological and genomic features of the lesion from which they originated [57]. Tumor-derived organoid libraries can be used to evaluate inhibitors and drug combinations before clinical trials [24,58,59,60]. The constitution of multicellular organoids could better represent normal organ and tumor biology. Ramamoorthy et al. established a multi-cellular lung organoid which mimicked the lung microenvironment with air sac-like structures and the production of lung surfactant proteins [61]. Singh et al. described how the use of 3D-derived organoids better evaluates treatment response to chemotherapy (doxorubicin), nanomedicine (Doxil^®^), biological therapy (Avastin^®^) and their combination when compared to independent cell suspension injection [62].

Clinical pilot trials aiming to validate tumoroids as a useful tool are already ongoing.

Ye Yao et al. implied in their study that patient-derived organoids could predict locally advanced rectal cancer patient chemoradiation responses, which emphasizes the fact that they can be a powerful tool in cancer treatment [63].

Angiospheres are more than just an in vitro model, as we were able to find similar structures in ascites from patients with EOC. It is well known that ovarian cancer cells detach from the tumor and float in the ascites as free-floating multicellular aggregates, called spheroids. However, to our knowledge, we are the first to show that endothelial cells could be present in those aggregates. The presence of Akt-activated ECs in ascites spheroids could provide an important advantage to cancer cells regarding their implantation on the peritoneal wall. In fact, we demonstrated ex vivo that spheroids with Akt-activated ECs were able to deeply invade peritoneal biopsies. The sprouting of ECs inside the peritoneum suggests early neo-vascularization by an activated endothelium. If confirmed in vivo, this could provide new insights into the intra-peritoneal spread of OCCs.

Our study once more highlights the emerging role of endothelial transmembrane and secretory growth factors and trophogens, referred to as angiocrine factors, as essential players in physiological processes and tumor growth. This newly emerged non-angiogenic role of tumor endothelia has greatly changed our perception of endothelium contribution to cancer biology and introduced new therapeutic potential for targeting cancer. However, selective targeting of angiocrine endothelia is still in its infancy and requires comprehensive understanding of the interactions occurring in the dynamic microenvironment of the tumor and its stroma.

## 4. Materials and Methods

### 4.1. Cell Cultures

Ovarian cancer cell line SKOV3 was purchased from ATCC and cultured following ATCC’s recommendations (ATCC, Manassas, VA, USA). A primary ovarian cancer cell line was derived in our laboratory from ascites of a patient with Stage III serous adenocarcinoma (APOCC) [21]. The cell lines were cultured in DMEM high glucose (Hyclone, Thermo Scientific, Waltham, MA, USA), 10% FBS (Hyclone, Thermo Scientific, Waltham, MA, USA), 1% Penicillin-Streptomycin-Amphotericyn B solution (Sigma-Aldrich Corp., St. Louis, MO, USA), 1X Non-Essential Amino-Acid (Hyclone, Thermo Scientific, Waltham, MA, USA) and 1% L-glutamine. Cultures were incubated in humidified 5% CO_2_ incubators at 37 °C and the media were replaced every 3 days [21].

We used our model of HUVECs with autonomous Akt activation surviving in the absence of FBS and cytokines (ECs) as a surrogate for tumor-associated endothelia [14,15,17,19]. E4orf1 transfected HUVECs (E4 + EC) were obtained as previously described [12]. HUVECs were purchased from ATCC and cultured following ATCC’s recommendations (ATCC, Manassas, VA, USA). Cells were cultured in endothelial cell growth medium (Medium 199, 20% (*v*/*v*) fetal bovine serum (FBS), 20 μg mL^−1^ endothelial cell growth supplement (Hallway), 1% (*v*/*v*) antibiotics (Hallway) and 20 units mL^−1^ heparin). In the E4 + EC model, the transfection of the adenoviral cassette E4orf1 in HUVECs provides low levels of Akt activation, allowing the use of serum-free, cytokine-free media without inducing immortalization or altering the endothelial phenotype [12,64].

### 4.2. Sphere Formation

PKH26 fluorescent cell linker (PE-conjugated) dye was purchased from Sigma (USA) and used according to the manufacturer’s instructions [18]. OCCs were initially stained with PKH26 dye and subsequently resuspended in 3D media containing DMEM F-12 supplemented with 2% B27 (Invitrogen), 20 ng/mL VEGF (Peprotech), 20 ng/mL bFGF (Peprotech) and 5 ug/mL insulin (Sigma). The cells were plated at a ratio of 1/3 (eGFP + E4 + ECs/OCCs) at 60000/20000 cells per well of ultra-low attachment 24-well plates (Costar, Corning) and were grown in a humidified incubator at 37 °C with 5% CO_2_. The media were changed every third day. Spheres were cultured for up to 5 days [15]. Fluorescence imaging was performed with an Evos^®^ FL digital inverted fluorescence microscope (AMG) or with a confocal microscope (see the confocal section).

### 4.3. Cell Proliferation Assay

Cells were plated at 50,000 cells per well in a 6-well plate in medium without FBS. Cells were then counted with a hemocytometer for the following six days every two days. Two wells were counted per condition. The experiment was performed in triplicate [65].

### 4.4. shRNA Transfection

Lentiviral particles containing shRNA against human Jagged1 (sc-37202-V), scrambled lentiviral particles (sc-108080) and polybrene (sc-134220) were purchased from Santa Cruz Biotechnology (Dallas, TX, USA). In summary, E4-ECs were cultured up to 50% confluence and were then treated with polybrene and lentiviral particles containing shRNA against Jagged1 or scrambled particles. Transfected cells were then selected using puromycin (Sigma, USA), and down-regulation of Jagged1 was assessed by qPCR [14].

### 4.5. Ascites and Peritoneal Sampling

Ascites and peritoneal samples were collected from patients included in the PELVIMASS protocol, which was accepted by the French Research Ethics Committee chair (CPP n° 2016-A01381-42). All patients provided written informed consent.

For ascites, patients with EOC presenting more than 10mL of ascites were included. Ascites were harvested at the moment of laparotomy. Normal peritoneal samples from patients with suspicious ovarian tumors that were ultimately found to be benign were used in this study. All conditions required surgical treatment with laparotomy. In each patient, 4 cm2 peritoneal samples were harvested at a required peritoneal incision site (broad ligaments) at the time of incision. Peritoneal samples were incubated at 37 °C in DMEM low glucose for 6 h. Media were subsequently filtered, aliquoted and stored at −80 °C [65].

We confirm that all methods were performed in accordance with relevant guidelines and regulations.

### 4.6. Immunohistochemistry

Immunohistochemistry (IHC) was performed at the Institut Mondor de Recherche Biomédicale (IMRB, Créteil, France). Tissue samples were embedded in paraffin and cut into 3 µm sections. First, the sections were processed for staining with HES (hematoxylin-eosin-saffron). The sections were then heated at 56 °C and deparaffinized by dipping the slides in xylene for 15 min. They were dehydrated by immersing them in a serial dilution of ethanol for 5 min and then rinsed with water. The sections were placed in EDTA pH 9 and heated in the microwave for 4.5 min at 750 W and then heated again for 4.5 min at 500 W. The Trilogy^®^ product (which combines the three pretreatment steps: deparaffinization, rehydration and unmasking) was added to the sections which were then heated again in the microwave at 750W for 5 min before then being rinsed in PBS/Tween. Primary antibody incubation was carried out using the Phospho-AKT (SER473) (D9E) XP Rabbit mAB kit overnight at 4 °C. After the overnight incubation, the secondary antibody (Impress rabbit) was incubated for 30 min and then washed several times. Slides were then revealed with DAB and counterstained with hematoxylin, rinsed with different washes (water, lithium carbonate) and dehydrated in different washes of ethanol and xylene. The sections were then ready for mounting. Akt expression was analyzed by measuring the percentage of Akt-positive areas and staining intensity. Akt expression was analyzed by measuring the Akt-positive ratio of the blood vessels.

### 4.7. Flow Cytometry

Fluorescence (FL) was quantified on a SORP FACSAria2 (BD Biosciences, Dubai, United Emirates) as previously described [15,66]. Data were processed with FACSDiva 6.3 software (BD Biosciences, Dubai, United Arab Emirates). Doublets were excluded by FSC-W × FSC-H and SSC-W × SSC-H analysis. eGFP fluorescence was acquired with a 488 nm blue laser with 510/50 nm emission. EpCam APC conjugate (BD Biosciences, Dubai, United Emirates) was acquired with a 647 nm red laser with 670/14 nm emission. PKH red fluorescence was acquired with a 535 nm green laser with 582/15 nm emission [67]. Charts display the median of fluorescence intensity (mfi) relative to control. Single-stained channels were used for compensation and fluorophore minus one (FMO) controls were used for gating. In total, 20,000 events were acquired per sample. Viability was assessed by flow cytometry evaluation of Calcein AM staining as described by the manufacturer (Live Dead Viability/Cytotoxicity Kit, Molecular Probes, Invitrogen) [67].

### 4.8. Confocal Microscopy

The interactions between PKH26 + OCCs and GFP + E4 + ECs in angiospheres were imaged using a Zeiss confocal Laser Scanning Microscope 710 (Carl Zeiss). Post-acquisition image analysis was performed with Zeiss LSM Image Browser Version 4.2.0.121 (Carl Zeiss). Spheres were imaged live using glass-bottom microwell plates (MatTek Corporation, Ashland, MA, USA) [65].

### 4.9. Western Blot Analysis

Western blots were conducted as previously described [68]. Immunostaining was performed using goat monoclonal antibodies against phospho-Akt (S473) (cell signaling #9271) and actin (1/1000, cell signaling) and a secondary polyclonal mouse anti-goat antibody HRP conjugate (1/2000, cell signaling). Blots were developed using HRP and chemiluminescent peroxidase substrate (#CPS1120, Sigma). Data were collected using a Geliance CCD camera (Perkin Elmer, Waltham, MA, USA) and analyzed using ImageJ software (NIH) [21].

### 4.10. Statistical Analysis

All quantitative data are expressed as mean ± standard error of the mean (SEM). Statistical analysis was performed by using SigmaPlot 11 (Systat Software Inc., Chicago, IL, USA). A Shapiro–Wilk normality test, with a *p* = 0.05 rejection value, was used to test normal distribution of data prior to further analysis [69]. All pairwise multiple comparisons were performed by one-way ANOVA, followed by Holm–Sidak post hoc tests for data with normal distribution or Kruskal–Wallis analysis of variance on ranks followed by Tukey post hoc tests in the case of a failed normality test. Paired comparisons were performed by Student’s *t*-tests or by Mann–Whitney rank sum tests in the case of unequal variance or a failed normality test. Statistical significance was accepted for *p* < 0.05 (*), *p* < 0.01 (**) or *p* < 0.001 (***). All experiments were performed in triplicate [70].

## Figures and Tables

**Table 1:** Human cytokine array performed on the supernatant of E4 + ECs and HUVECs treated or not treated with LY294002 and/or FGF2. The table displays the pixel density of the dots for each cytokine.

	CELLS				CELLS			
	HUVEC Ctrl	HUVEC FGF	HUVEC LY	HUVEC FGF LY	E4 Ctrl	E4 FGF	E4 LY	E4 FGF LY
Activin A	23.6	27.2	13.1	0	26.3	17.2	0	0
ADAMTS-1	11.5	14.7	0	0	18.8	11.9	0	0
Angiogenin	18.2	27.8	0	0	19.9	13.0	0	0
Angiopoietin-1	27.3	29.0	12.3	0	19.2	16.4	0	0
Angiopoietin-2	63.8	55.3	34.0	26.0	18.0	20.3	0	0
Angiostatin	25.9	19.8	0	0	13.9	16.6	0	0
Amphiregulin	22.9	16.5	0	0	14.1	16.1	0	0
Artemin	32.6	24.8	14.6	11.2	18.1	22.8	0	0
DPPIV	27.1	31.4	16.8	0	97.0	72.6	30.0	34.6
EGF	10.7	13.0	0	0	22.0	13.6	0	0
EG-VEGF	32.5	38.0	14.5	12.2	24.5	14.7	0	0
Endoglin	150.5	138.1	112.0	90.5	112.5	102.9	51.7	46.3
Endostatin	91.2	86.7	58.5	47.6	53.5	48.6	0	0
Endothelin-1	92.6	82.6	71.9	61.3	43.8	41.4	0	12.1
FGF acidic	63.3	32.4	45.9	23.3	53.0	49.6	17.3	10.7
FGF basic	107.1	139.6	103.2	115.6	144.4	179.1	96.1	91.3
FGF-4	21.7	14.1	0	0	25.0	34.2	0	0
FGF-7	20.3	13.9	0	0	13.7	12.6	0	0
HB-EGF	27.7	33.5	15.8	14.3	34.8	16.1	0	10.6
HGF	14.3	15.1	0	0	19.8	0	0	0
IGFBP-1	30.9	34.7	12.7	12.8	25.2	17.9	0	0
IGFBP-2	25.5	26.6	0	0	22.3	16.3	0	0
IGFBP-3	32.9	29.6	13.2	0	21.8	18.9	0	0
IL-1β	24.2	24.9	13.3	0	17.9	20.9	0	0
IL-8	90.4	108.2	114.6	91.2	79.8	88.8	43.2	37.8
LAP (TGF-β1)	59.1	49.4	33.8	28.9	50.7	64.9	17.3	13.2
Leptin	21.9	19.4	18.8	11.8	15.8	22.4	0	0
MCP-1	28.3	22.3	10.7	0	13.7	16.1	0	0
MMP-8	20.9	25.7	20.4	10.6	15.8	10.7	0	15.3
MMP-9	18.0	21.8	0	0	17.5	0	0	0
NRG1-β1	16.9	21.5	0	0	20.9	12.6	0	0
Pentraxin 3 (PTX3)	108.2	121.5	64.0	55.1	135.7	128.3	54.6	45.9
PD-ECGF	25.3	29.9	14.6	11.1	28.9	31.2	0	0
PDGF-AA	27.3	30.6	15.7	10.3	20.9	25.4	0	0
PDGF-AB/PDGF-BB	29.4	42.5	27.6	26.0	23.4	29.6	0	0
Persephin	39.4	35.2	23.2	18.2	22.3	37.1	0	0
Platelet Factor 4 (PF4)	23.4	18.3	10.1	0	16.9	26.8	0	0
PIGF	108.9	146.1	117.3	105.3	30.0	35.5	15.3	0
Prolactin	20.1	18.2	0	0	10.9	12.8	0	0
Serpin B5	21.5	24.8	21.4	0	0	10.4	0	0
Serpin E1	180.2	193.0	181.4	158.2	146.6	172.4	122.0	132.1
Serpin F1	15.8	21.8	0	0	14.0	11.9	0	0
TIMP-1	80.4	117.4	48.6	52.8	122.0	122.8	22.2	44.3
TIMP-4	23.5	32.3	10.8	0	26.0	22.3	0	0
Thrombospondin-1	128.6	141.9	113.8	97.0	108.7	119.8	58.2	62.9
Thrombospondin-2	24.5	29.9	18.1	11.3	20.7	27.6	0	0
uPA	170.7	201.4	171.3	151.6	179.0	206.6	92.4	101.8
Vasohibin	24.2	22.0	12.9	0	28.2	47.0	0	0
VEGF	19.2	16.4	0	0	13.1	19.4	0	0
VEGF-C	21.6	21.6	0	0	0	15.6	0	0

## Data Availability

All data that support the findings of this study are available from the corresponding authors upon reasonable request.

## Supplementary Materials

The following supporting information can be downloaded at: https://www.mdpi.com/article/10.3390/ijms232214173/s1.

## List of Abbreviations

APOCC: ascites primary ovarian cancer cells
bAB: binding antibody
BM: bone marrow
E4 + ECs: Akt-activated endothelial cells transfected with E4ORF gene
EC: endothelial cell
EOC: epithelial ovarian cancer
ERK: extracellular signal-regulated kinases
FGF2: fibroblast growth factor
GSI: gamma secretase inhibitor
HSC: hematopoietic stem cell
HUVEC: human umbilical vein endothelial cell
OCC: ovarian cancer cell
pAkt: phospho-Akt
PD-ECGF: platelet-derived endothelial cell growth factor
PFS: progression free survival
PTX3: Pentraxin 3
TIMP-1: tissue inhibitor of metalloproteinases-1
TM: tumor microenvironment
VEGF: vascular endothelial growth factor
