# Supplementary material for: Akt-Activated Endothelium Increases Cancer Cell Proliferation and Resistance to Treatment in Ovarian Cancer Cell Organoids
Source: Int J Mol Sci. 2022 Nov 16;23(22):14173. doi: 10.3390/ijms232214173 (PMC9694384; doi:10.3390/ijms232214173)
Supplement: Supplementary file 1 [file ijms-23-14173-s001.zip › ijms-1819542-SI.pdf]

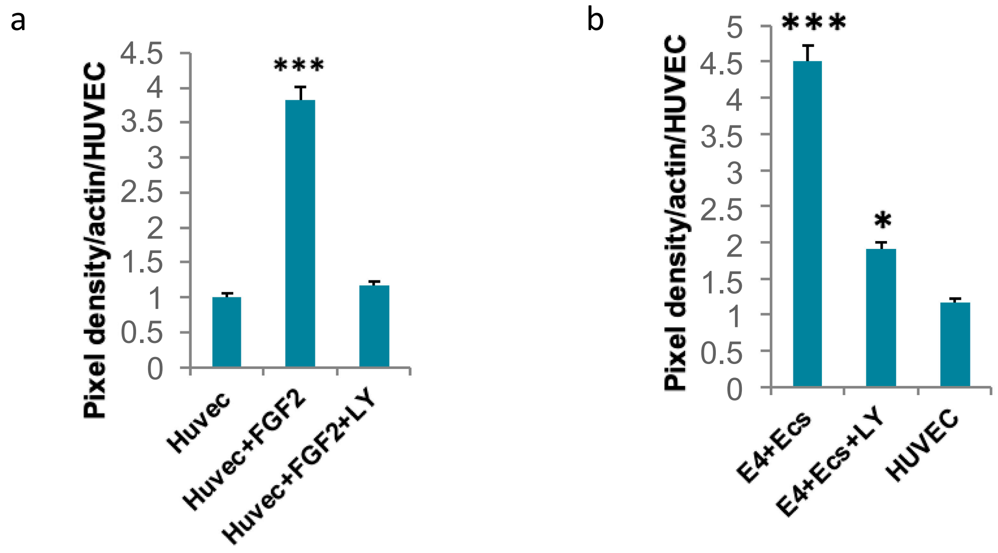

Figure S1. Western blot quantification for figures 1E (**a**) and 1H (**b**)
Bar chart shows quantification of pAKT compared to actin, 1 is the pixel density of HUVEC. $p < 0.05$ (*), $p < 0.001$ (***).

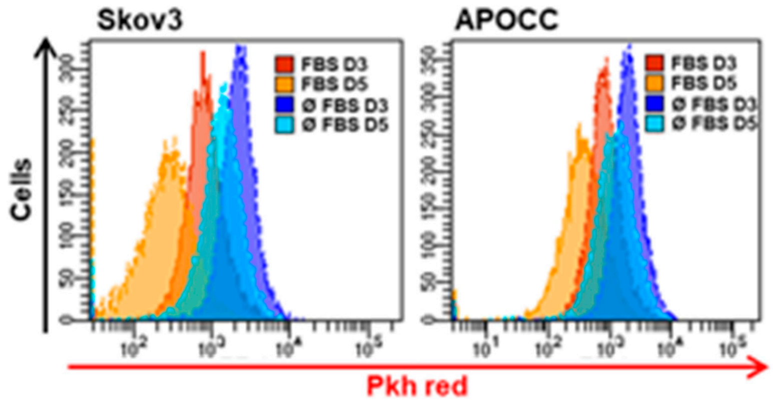

Figure S2. OCC (SKOV3 and APOCC) were stained with PKH red prior culture with or without FBS. PKH staining intensity was evaluated at D3 and D5 in flow cytometry.

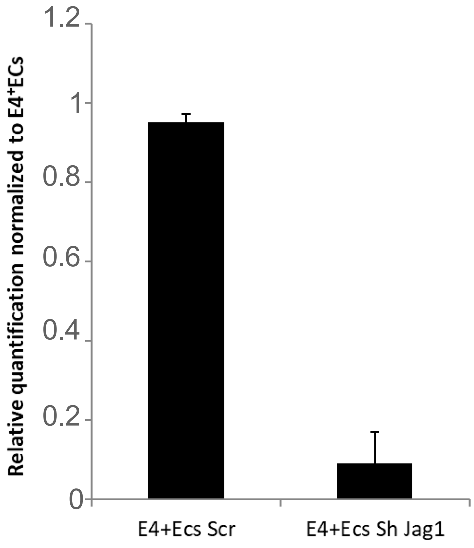

Figure S3. qPCR for jag1 in E4+ECs scrambled and E4+ECs SH for Jag1.

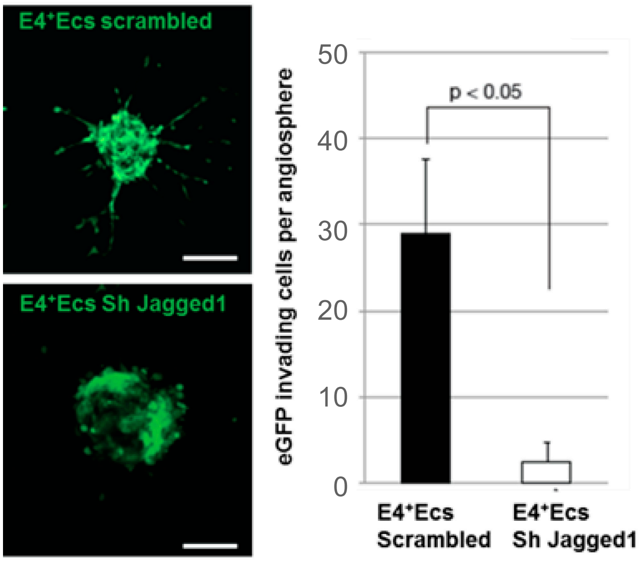

Figure S4. eGFP-E4+ECs scrambled and eGFP-E4+ECs SH for jag1 were co-cultured in 3D media as sphere with SKOV3. Endothelial tube formation intra-sphere was evaluated through eGFP fluorescence in confocal. Scale bar, 50 µm.

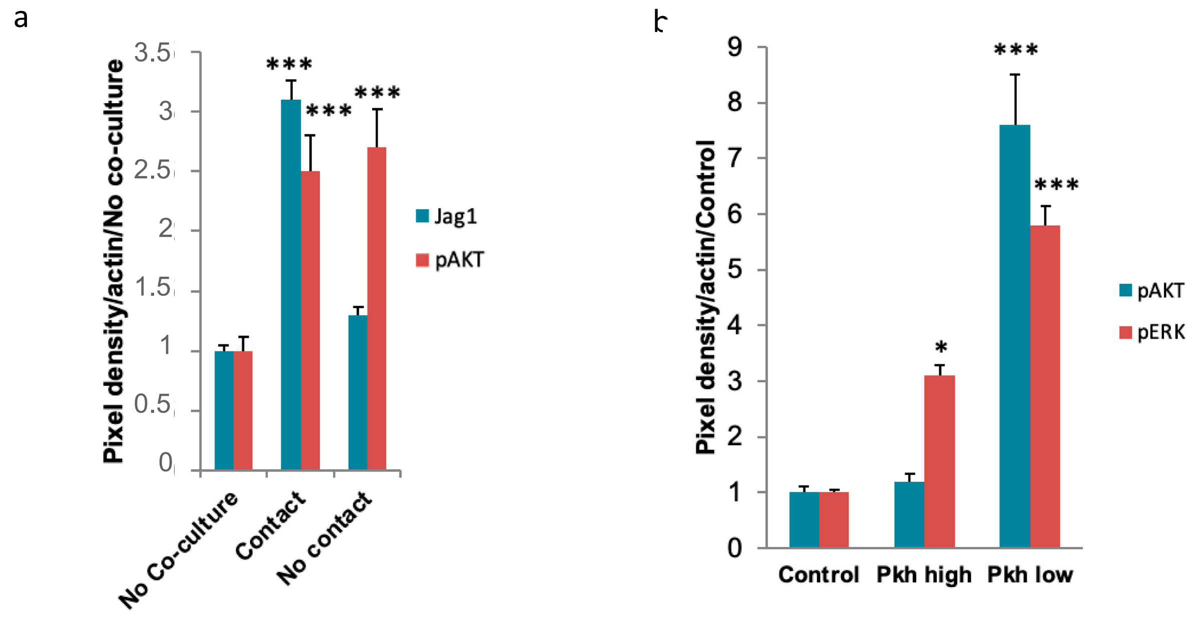

Figure S5. Western blot quantification for figures 2E (**a**), 2I and 2J (**b**)
a Bar chart shows quantification of Jag1 and pAKT compared to actin, 1 is the pixel density of No co-culture **b** Bar chart shows quantification of pAKT and pERK compared to actin, 1 is the pixel density of control. $p < 0.05$ (*), $p < 0.001$(***).

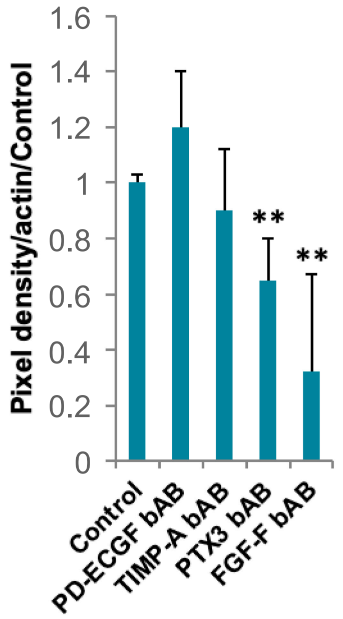

Figure S6. Western blot quantification for figure 3C: Bar chart shows quantification of pAKT compared to actin, 1 is the pixel density of control. $p < 0.01$ (**).

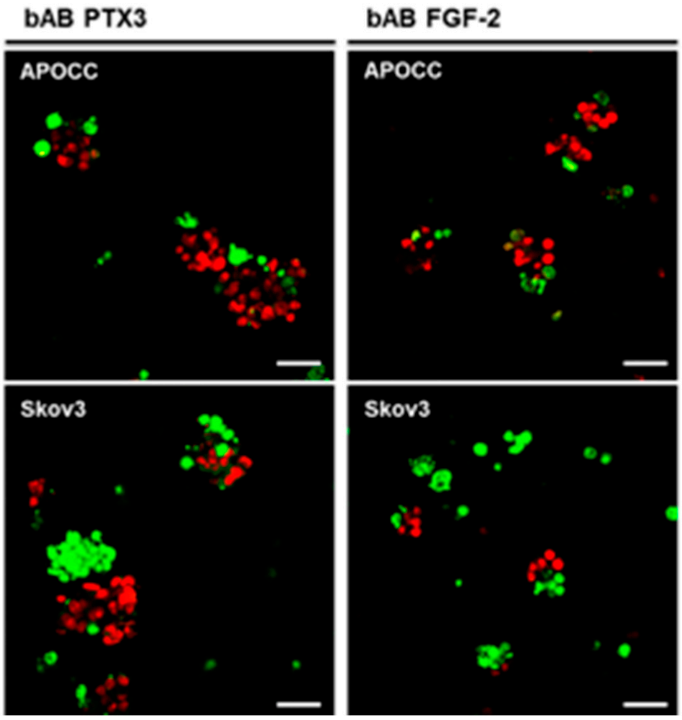

Figure S7. Spheroids of OCCs (SKOV3 and APOCC) were stained with PKH red prior co-culture in 3D media with eGFP-E4+ECs. Spheroids were cultured for 6 days in presence of different blocking antibodies (PTX3 bAB, and FG2 bAB). Confocal imaging was performed on the spheroids. Scale bar, 50 µm.

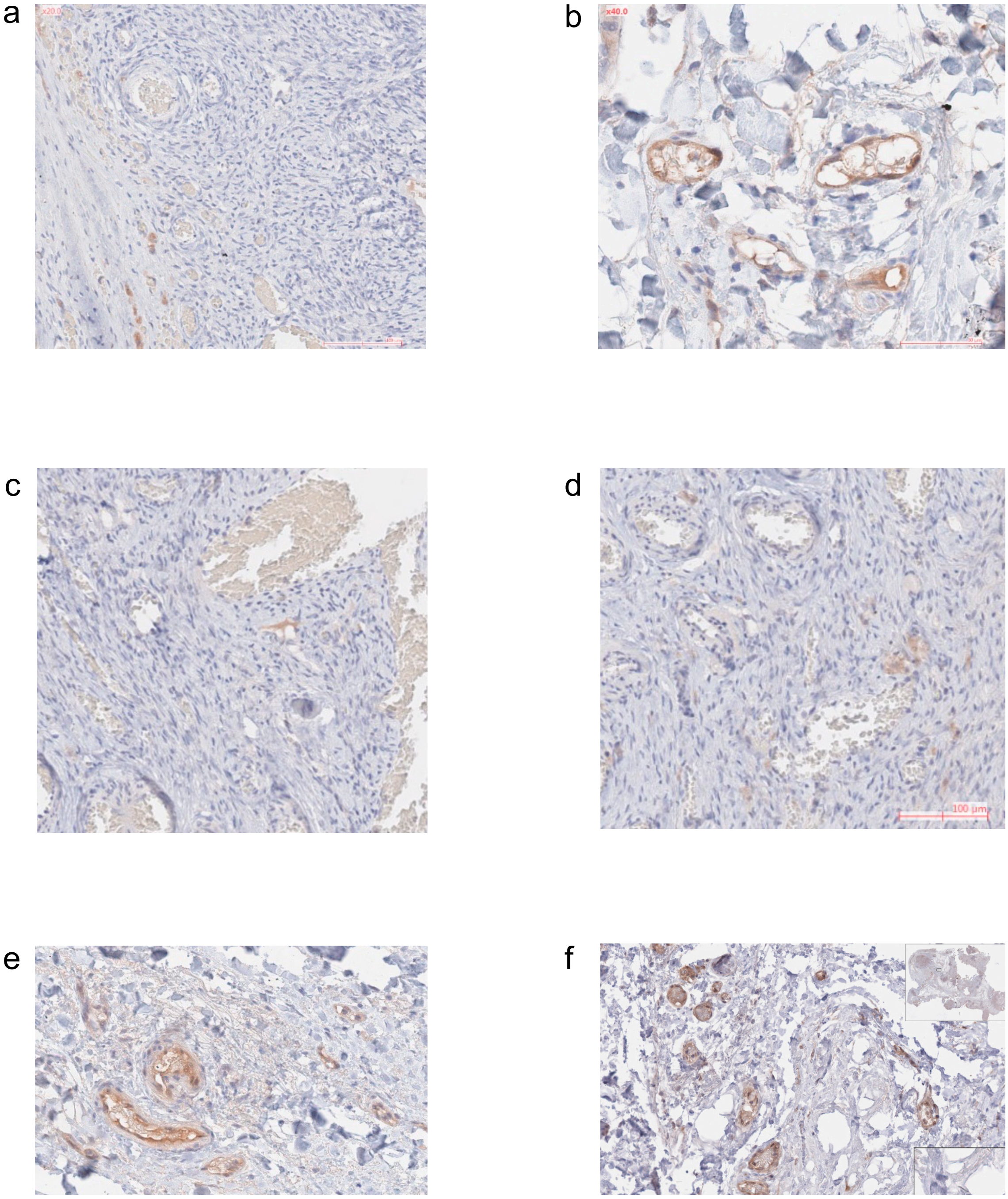

Figure S8. Representative immunohistochemistry images for p-Akt expression in normal ovary (**a**), benign serous (**b**), serous borderline tumor (**c**), high grade serous pre-chemotherapy carcinoma (**d**), high grade serous carcinoma (**e**) and high grade serous post-chemotherapy carcinoma (**f**)

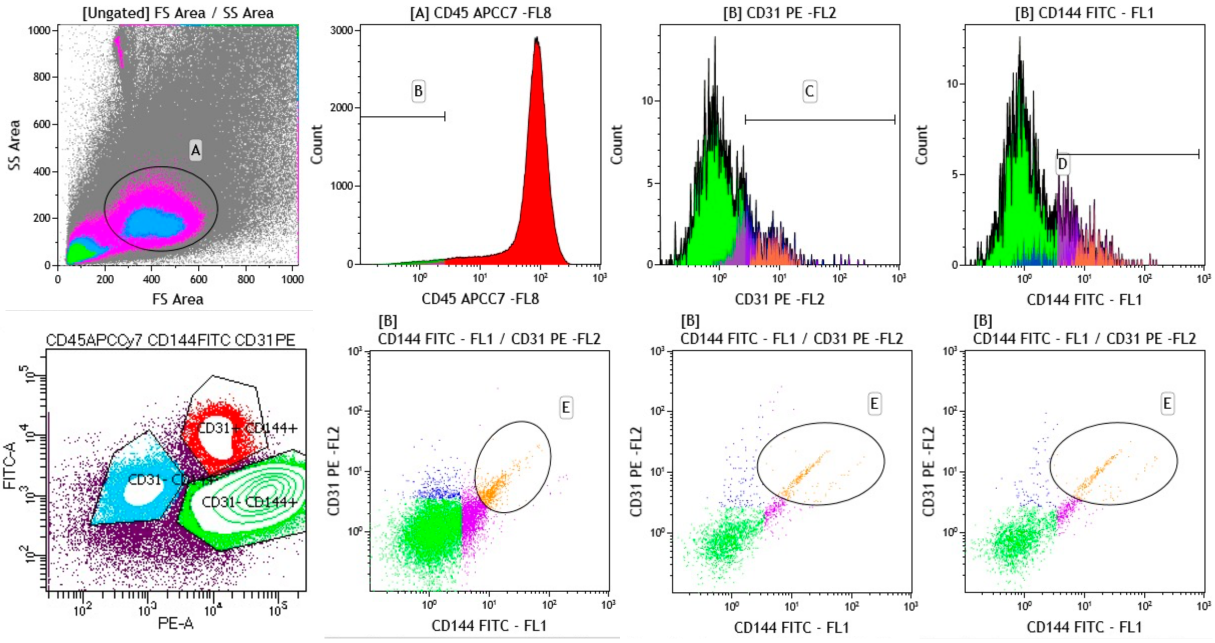

CD31$^+$/CD44$^+$
-In all pop: 1.20%
-In CD45$^-$ pop: 5.90%

CD31$^+$/CD44$^+$
-In all pop: 1.04%
-In CD45$^-$ pop: 7.74%

CD31$^+$/CD44$^+$
-In all pop: 1.26%
-In CD45$^-$ pop: 4.51%

CD31$^+$/CD44$^+$
-In all pop: 1.03%
-In CD45$^-$ pop: 8.14%

Figure S9. Gating strategy for the detection of endothelial cells in patient ascites. Gating strategy for the detection of endothelial cells in patient ascites. A. Gating of living cells. B. Gating of CD45- on living cells (Gate A). C. Gating of CD31+ inside living cells that are CD45- (Gate B). D. Gating of CD144+ cells inside living cells that are CD45- (Gate B). E. CD31+/CD144+ cells inside living cells that are CD45- (Gate B).
